# Mental Health of Chinese People During the COVID-19 Pandemic: Associations With Infection Severity of Region of Residence and Filial Piety

**DOI:** 10.3389/fpsyg.2021.633452

**Published:** 2021-05-28

**Authors:** Wendy Wen Li, Yahong Li, Huizhen Yu, Dan J. Miller, Christopher Rouen, Fang Yang

**Affiliations:** ^1^College of Healthcare Sciences, James Cook University, Townsville, QLD, Australia; ^2^Department of Applied Psychology, South Central University for Nationalities, Wuhan, China; ^3^Department of Social Work, Foshan University, Foshan, China

**Keywords:** filial piety, stress, anxiety, depression, COVID-19 infection severity, mental health prevalence, terror management theory, meaning maintenance model

## Abstract

This study aims to investigate mental health among Chinese people living in areas with differing levels of infection severity during the COVID-19 outbreak. It also assesses the association between reciprocal and authoritarian filial piety and mental health in times of crises. A sample of 1,201 Chinese participants was surveyed between April and June 2020. Wuhan city (where 23.4% of participants resided), Hubei province outside Wuhan (13.4% of participants), and elsewhere in China (63.1% of participants) were categorized into high, moderate, and low infection severity areas, respectively. The Depression, Anxiety, and Stress Scale’s severity cut-points were used to categorize participants. In the overall sample, 20.9, 34.2, and 29.0% of the participants showed elevated (mild to extremely severe) levels of stress, anxiety, and depression. Those in the highest infection severity group were significantly more likely to be categorized as having elevated levels of stress, anxiety, and depression. General linear modeling was performed on a composite mental distress variable (taking into account stress, anxiety, and depression scores). This model indicated that, even after adjusting for group differences in age, gender, education, and filial piety, the high infection severity group displayed more mental distress than the low infection severity groups. The model also found reciprocal filial piety to have a negative association with mental distress. Conversely, authoritarian filial piety was found to be unrelated to mental distress when controlling for the other variables in the model. No evidence was found for an interaction between either authoritarian or reciprocal filial piety and infection severity, which suggests that the negative association observed between reciprocal filial piety and mental distress was relatively consistent across the three infection severity groups. The findings suggest that future public health programs may integrate the promotion of filial piety as a strategy to help Chinese people maintain good mental health in the face of pandemic crises.

## Introduction

### Background

By November 15, 2020, the novel coronavirus (COVID-19) pandemic affected 220 countries and territories around the world, with 53,766,728 confirmed cases and 1,308,975 confirmed deaths (World Health Organization (WHO), 2020). The pandemic has impacted many people in unprecedented ways, including grave departures from their normal lives. Millions have been required to come to terms with the profound loss of loved ones, experiencing fear, anxiety, and depression. The people of Wuhan, Hubei province, China—where the COVID-19 outbreak was first noted, and which has had the highest number of confirmed cases and deaths in China—experienced collective bereavement and grief (Cao Y. et al., 2020) when their city was forced into complete lockdown for 76 days. Moreover, the constantly changing health alerts and overwhelming media coverage of the spread of COVID-19 within the city escalated fear, anxiety, and even stigma among city residents, all of which may have profound impacts on mental health. As Wuhan was the most severely affected area in China, it is reasonable to expect that the mental health of Wuhan residents may have been more severely negatively impacted compared with those living in less severely affected cities.

The COVID-19 pandemic has intensified people’s awareness of death and death-related anxiety (Li et al., 2020c). The theoretical underpinnings of terror management theory (TMT) and the meaning maintenance model (MMM) consider the impact of death-related anxiety and reach similar conclusions regarding the importance of culturally salient relationship models. Although not tested in this study, these two theories are employed to guide the interpretation of results. According to TMT, human awareness of the inevitability of death may generate death-related anxiety, which is aversive and disruptive to psychological functioning (Pyszczynski et al., 2015). Empirical evidence suggests that COVID-19 outbreaks are likely to lead to mental health crises, especially in areas with high numbers of confirmed cases and deaths (Dong and Bouey, 2020), such as Wuhan. In a survey on the psychological impact on the general population of China within the first 2 weeks of the COVID-19 outbreak, Wang et al. (2020) found that 53.8% of 1,210 participants reported that the pandemic had had a moderate to severe psychological impact on them. The study of Xiao et al. (2020a) on the mental health and sleep quality of 170 participants who self-isolated at home for 14 days in central China similarly found participants’ anxiety and stress to be high, while their sleep quality was low.

Several studies have investigated differences in mental health between people living in areas that are severely infected by a pandemic, compared with those in less affected areas. Lau et al.’s (2008) study on the impact of the Severe Acute Respiratory Syndrome (SARS) outbreak in Hong Kong in 2003 found people living in areas with a high incidence of SARS cases showed significantly lower levels of subjective wellbeing, particularly among participants who were elderly, female, or less educated. People living in areas with a higher incidence of infection are likely to have a more pronounced fear of infection compared with their counterparts in mildly infected areas. Studies on previous pandemics have also shown that the greater the scale of the outbreak, the greater the fear and anxiety experienced and, thus, the larger the impact on mental health (Shultz et al., 2016). Fear, as a motivational state aroused by a pandemic, may give rise to defensive behaviors (Steimer, 2002), which may trigger emotional and behavioral contagions (Shultz et al., 2016). During a pandemic, emotional contagion is the spread of fearful mood and negative affect through the population. Behavioral contagion is the tendency for certain behaviors exhibited by one person to be copied by others (Duan et al., 2019). Both emotional and behavioral contagions are likely to escalate pandemic-related fear, arousing negative affective responses to the pandemic at both the individual and collective level, thereby increasing the risk for psychological distress (Shultz et al., 2016). As a result, it is possible that, in high infection areas, the number of people feeling distress may be far greater than the number of people who are actually infected (Ornell et al., 2020) as greater levels of awareness of mortality increases fear of infection and death anxiety. TMT holds that the fear and anxiety caused by the awareness of death can be managed through an anxiety-buffering system (Jonas et al., 2014; Maxfield et al., 2014). The anxiety-buffering system includes two main defense mechanisms: strengthening self-esteem and promoting cultural worldviews. These defense mechanisms can be drawn on, when human awareness of the inescapability of death becomes salient, to help individuals maintain mental health (Greenberg et al., 1986; Solomon et al., 1991; Jonas et al., 2014). Cultural values such as filial piety may therefore act to buffer death-related anxiety for Chinese people. The Confucian concept of filial piety refers to moral norms and practices of respect and caring for one’s parents. Not only does it require filial duties such as material and emotional support to, and co-residence with, one’s parents (Li et al., 2010) but also compliance and obedience to parental demands (Li, 2013; Bedford and Yeh, 2019). It ascribes the ideal relationship between parent and child, which places the family at the core of the Chinese moral worldview (Li, 2013; Li and He, 2019).

In contrast to the Christian view of the ontology of the universe, Confucianism does not advocate a transcendent creator. Instead, Confucianism posits that one’s life is an extension of his/her parents’ physical lives (Li, 2013). Hence, a person exists solely because of his/her parents. This notion upholds that the greatest gift children receive from their parents is life itself (Sung, 1995). Children’s commitments to deferring to their parents’ wishes, attending to their parents’ needs, and providing care and support to their parents are reciprocal to the gift of life. Confucianism further conceptualizes a family being one body; and one should not harm one’s own body in any situation (Hwang, 1999). In the context of a pandemic, Chinese people are likely to adopt a proactive strategy to protect their parents and family members, which is evident in empirical research. Wills and Morse’s (2007) study on Canadian Chinese people’s responses to the threat of SARS found that participants’ reaction to SARS was strongly influenced by filial piety; as reflected in participants’ desires to protect their family members and community by strictly following the government’s instructions and taking all efforts to ensure that they did not spread the virus. In this case, filial piety moved beyond the filial obligation of showing respect to one’s parents, to a moral principle that directed responses to protect one’s community (Wills and Morse, 2007).

Filial piety reflects the five cardinal ethics for the five major dyadic relationships proposed by Confucianism: the affective relationship between father and son; righteousness between sovereign and subordinate; distinction between husband and wife; proper order between older and younger brothers; and trustworthiness between friends (Li et al., 2020a). These relationships, apart from the relationship between friends, are characterized as vertical and authoritarian between sovereign and subordinate (Hwang, 1999). The authoritarian nature of the relationships is internalized *via* moral education, which encourages the subordinate to believe that the sovereign’s decisions are consistent with the tenets of righteousness, justice, and compassion; and that subordinates are socially and morally expected to display faithfulness, compliance, and obedience toward the sovereign (Ho, 1996; Hwang, 1999; Li et al., 2020a). The hierarchical structure of authority within the family unit, ascribed by filial piety, has been extended beyond the household regime, and applied to authority relationships in society more generally (Yeh and Bedford, 2003; Bedford and Yeh, 2019); termed “parallel filial piety of society” (Kutcher, 1999; Li, 2013). The concept of parallel filial piety of society suggests that broader societal relations parallel family relations. In other words, filial Chinese individuals should view their obligations to their parents as essentially parallel to their obligations to authority. This concept reflects the Confucian political philosophy that encourages citizens to transform filial devotion for their parents to loyalty to authority, which results in people’s attitudes toward their parents being mirrored in their attitudes toward authority (Kutcher, 1999). This notion is evident in contemporary Chinese culture. For example, Li et al. (2020c) maintains that China being a “dear mother” is a common motif in contemporary Chinese popular culture (e.g., music and literature), representing the cultural belief that the state is a symbolic parental figure for Chinese people, and reinforcing the cultural expectation of being filial to authority.

The COVID-19 pandemic makes cognizant the concept of death and its inevitability, instilling existential terror in some. According to TMT (Greenberg et al., 1986; Pyszczynski et al., 2015) an important function of cultural worldviews is to mitigate existential terror. For example, TMT would posit that filial piety, a culture-specific worldview for Chinese people, may assuage anxiety by providing standards of value that are derived from the cultural belief of an authoritarian relationship between subordinate and sovereign (which facilitates the subordinate to prosper into a filial and responsible self who follows the moral and social standards set by the sovereign), and by promising protection from being infected, and death transcendence, to those who develop a pro-quarantine attitude, stringently obey role obligations, and voluntarily sacrifice their personal wishes for the collective good of the nation.

Meaning maintenance model (Heine et al., 2006) has similarly been employed to offer an understanding of how people respond to fear associated with mortality and the unprecedented existential threat caused by COVID-19 (Li et al., 2020c). MMM proposes that people innately and automatically assemble mental representations of expected relational systems (which they strive to make coherent and consistent), leading to a sense of symbolic unity. The theory posits that human beings are meaning makers, capable of attributing meaning through actively building new connections and coherent relations, especially when the sense of symbolic unity that these relations provide is disrupted. As meaning makers, people do not submissively react to meaning violations, where coherent relations and one’s sense of meaning is threatened by an unexpected crisis, such as the COVID-19 pandemic (Heine et al., 2006; Proulx and Inzlicht, 2012). The COVID-19 pandemic has disrupted people’s relationships and interactions as a result of lockdown. Threats to the expected relational systems motivate people to regain meaning by reconstructing order, normality and certainty, and re-establishing a sense of coherence (Proulx and Heine, 2006). The COVID-19 pandemic also threatens certainty. People encounter psychological distress concerning this uncertainty and endeavor to cope with this. The need to reduce uncertainty leads individuals to actively recognize meaning violations and, consequently, reformulate meaning *via* a reconstruction of coherent relations, meaningful associations and the sense of symbolic unity.

Situating the reaffirmation of relational meaning in the Chinese cultural context connects MMM with filial piety. Filial piety maintains that a person’s life is meaningful only through coexistence with others and a series of obligations between people in different relationships (Hwang, 2001; Li and Forbes, 2018; Li et al., 2020a). It emphasizes coherent and meaningful social relations that contribute to the greater good of the public, which reflects MMM’s central claim that meaning is relational. According to Heine et al. (2006), meaning is the expected relationship through which people construct and experience their world. People search for coherent relations within the environment they live, within themselves, and between themselves and the environment. During the COVID-19 pandemic, expected relationships may be interrupted. The perceived breakdown of these relationships may provoke people’s efforts to reconstruct meaningful relations and connectedness, and in turn, protect people’s psychological wellbeing. In the process, filial piety may assist individuals to restore meaning through reaffirming the relationships with their parents and the state.

Built upon the reciprocal and authoritarian natures of filial piety, the dual-factor model of filial piety (measured as part of the current study), posits that filial piety consists of two dimensions: reciprocity and authoritarianism (Yeh, 2003). Reciprocal filial piety is concerned with genuine affection that is developed through constructive relationship with one’s parents. People with attitudes that reflect reciprocal filial piety tend to attend to their parents’ needs out of gratitude. Similarly, they may support the state in exchange for the protection provided by the state. Authoritarian filial piety involves obedience to social expectations and suppression of one’s own desires to comply with the wishes of the parent or the state (Yeh, 2009). Reciprocal filial piety is stimulated by the psychosocial need for interpersonal relationships and social connections, whereas authoritarian filial piety is driven by the need for collective identification (Chen et al., 2016). Reciprocal and authoritarian filial piety are not mutually exclusive. Rather, they are interwoven and operate simultaneously in varying degrees, depending on circumstances (Bedford and Yeh, 2019). For example, the effect of authoritarian filial piety may be more significant than that of reciprocal filial piety within the context of the COVID-19 crisis, where people are expected to diligently follow the government’s health directives. Conversely, reciprocal filial piety may play a greater role in maintaining mental health because people feel that their personal sacrifices in the efforts to stop the spread of COVID-19 are adequately recognized and reciprocated by their parents and the state.

Previous research into the correlation between filial piety and wellbeing and mental health has found reciprocal filial piety to be negatively correlated with perceived depression and anxiety (Yeh, 2006); and positively associated to life satisfaction (Leung et al., 2010; Chen, 2014), social competence (Leung et al., 2010), subjective happiness and quality of family life (Chen et al., 2016), and mental wellness (Jen et al., 2019). Conversely, authoritarian filial piety has been found to be positively associated with perceived depression and anxiety (Yeh, 2006) and negatively correlated with self-esteem and social competence (Leung et al., 2010), and mental wellness (Jen et al., 2019). These studies suggest that reciprocal filial piety is a positive predictor of wellbeing and mental health, while authoritarian filial piety may have a negative influence on individual wellbeing and mental health.

### The Present Study

The present study aims to investigate the mental health of Chinese people living in high, moderate, and low infection severity areas during the COVID-19 pandemic, as well as assess filial piety’s association with Chinese people’s mental health in the time of COVID-19. To the authors’ knowledge, there are no extant studies comparing the mental health of people living in areas with different levels of COVID-19 infection severity, while also assessing the role of filial piety. Understanding the role of cultural factors (e.g., filial piety) in maintaining mental health in the time of a pandemic is significant. Culture is perhaps less visible compared with economic dynamics or the political processes at play during the COVID-19 pandemic. Nonetheless, cultural factors may contribute greatly to helping individuals maintain mental health during the global fight against COVID-19.

As previously mentioned, the mental health of people in areas with a greater spread of COVID-19 infection may be expected to be worse compared with those in less affected areas. Additionally, it might be expected that those with higher levels of filial piety will obey role obligations and sacrifice their personal wishes for the collective good of the family and nation, which may contribute to the maintenance of their mental health, regardless of whether they live in a high or low infection severity area. Alternatively, it is also possible that an association between filial piety and mental health would be present among those in low infection severity areas, but that this relationship would not be present among those living in areas with more extreme infection severity (i.e., the extreme situation may cause high levels of existential anxiety and overwhelm the potential protective effects of filial piety). As such, the interaction between filial piety and severity of infection on people’s mental health is also worth considering.

In the current study, three mental health-related outcome variables are assessed: stress, anxiety, and depression. Due to the exploratory nature of the current study, research questions, rather than *a priori* hypotheses, are employed. Three research questions are investigated:

1. Is infection severity predictive of mental health?
2. Are reciprocal and authoritarian filial piety predictive of mental health?
3. Is there an interaction between infection severity and filial piety in predicting mental health?

## Materials and Methods

### Participants

A total of 1,220 participants aged 18 years and over, who lived in the high, moderate, and low infection severity areas, were surveyed during the period of April 10 to June 10, 2020. Data cleaning (see below) left a final *N* of 1,201. In the final sample, 23.4% (*n* = 281) participants were categorized as living in a high infection severity area (Wuhan city), 13.4% (*n* = 162) were living in a moderate infection severity area of nine cities in Hubei province outside of Wuhan, with the remaining 63.1% (758) living in a low infection severity area of 97 cities elsewhere in China. The demographic characteristics of the final sample are reported in Table 1.

**TABLE 1:** Participant demographic characteristics.

**Variable**	**Mean (standard deviation)**
Age	29.62 (12.72)

	**Percentage (frequency)**

**Gender**	
Male	36.5 (438)
Female	63.5 (763)
**Highest level of formal education**	
Intermediate school	2.6 (31)
High school	5.8 (70)
Diploma	9.0 (108)
Undergraduate education	62.5 (751)
Postgraduate education	20.0 (241)
**Occupation**	
Public servant	3.7 (45)
Healthcare worker	7.8 (94)
Teacher/public institution employee	12.6 (151)
College student	52.5 (630)
Enterprise employee	5.7 (69)
Farmer	0.4 (5)
Factory worker	0.8 (10)
Retiree	3.3 (40)
Unemployed	4.3 (52)
Other	8.7 (105)

### Measures

#### Demographic Variables

Several demographic variables were assessed, including residential area during the COVID-19 pandemic, age, gender, highest level of education, and occupation.

#### Infection Severity

Infection severity in the current study refers to the severity of infection in the specific geographic area in which an individual resides, as opposed to the severity of an individual’s infection. Infection severity in region of residence was indexed based on the number of confirmed cases and deaths in the area a participant resided on April 16, 2020. According to the statistics provided by The National Health Commission of China (2020), as of April 16, 2020, there were 50,333 confirmed cases and 3,869 deaths in Wuhan (located in Hubei province). In Hubei province outside of Wuhan, there were 17,795 confirmed cases and 643 deaths. Elsewhere in China, there were 14,564 confirmed cases and 120 deaths. Thus, Wuhan, Hubei province outside of Wuhan, and elsewhere in China, were categorized as high, moderate, and low infection severity areas, respectively.

#### Mental Health Outcomes

Mental health outcomes were assessed utilizing the 21-item standardized Chinese version of the short Depression, Anxiety, and Stress Scale (C-DASS21; Taouk et al., 2001). This self-report questionnaire elicits scores for depression, anxiety, and stress using four-point scales where *0* = *did not apply to me at all* and *3* = *applied to me very much, most of the time*. Higher scores correspond to higher levels of depression, anxiety, and stress. Example items for stress include “I found it hard to wind down” and “I tended to over-react to situations.” Example items for anxiety include “I was aware of dryness of my mouth” and “I experienced breathing difficulty (e.g., excessively rapid breathing, breathlessness in the absence of physical exertion).” Items for depression include “I couldn’t seem to experience any positive feeling at all” and “I felt that I had nothing to look forward to.” To ensure consistency with scores on the 42-item DASS (and so that the cut-off scores established for the 42-item DASS could be employed), subscale scores were totaled and multiplied by two (Lovibond and Lovibond, 1995). Table 2 displays the cut-off values for stress, anxiety, and depression given for the 42-item DASS. These cut-points were developed based on a large, non-clinical sample of Australians (Lovibond and Lovibond, 1995). The scale’s authors indicate that it is permissible to create a composite measure of “negative emotional symptoms” by summing stress, anxiety, and depression scores (Lovibond and Lovibond, 1995). This composite score has a possible range of 0–126, with higher scores indicating more mental distress. The Chinese DASS has demonstrated good internal consistency in recent studies (Li et al., 2020b,c; Xie et al., 2021), with Cronbach’s alphas between 0.83 and 0.87, 0.78 and 0.87, and 0.83 and 0.88 for stress, anxiety, and depression, respectively. In the current study, Cronbach’s alphas for stress, anxiety, and depression were 0.90, 0.87, and 0.90, respectively.

**TABLE 2:** Cut-off scores used for each DASS subscale.

**Category**	**Stress**	**Anxiety**	**Depression**
Normal	0–14	0–7	0–9
Mild	15–18	8–9	10–13
Moderate	19–25	10–14	14–20
Severe	26–33	15–19	21–27
Extremely severe	34+	20+	28+

#### Filial Piety

Filial piety was measured by the standardized Chinese Dual Filial Piety Scale (Yeh and Bedford, 2003). The 16-item scale produces totals for reciprocal and authoritarian filial piety (eight items for each subscale) using a six-point scale in which *1* = *Extremely unimportant* and *6* = *Extremely important*. Sample items for reciprocal filial piety include “talk frequently with my parents to understand their thoughts and feelings” and “be concerned about my parents, as well as understand them.” Sample items for authoritarian filial piety include “take my parents’ suggestions even when I do not agree with them” and “let my income be handled by my parents before marriage.” Higher scores indicate greater reciprocal and authoritarian filial piety. The scale has previously demonstrated good internal consistency with Cronbach’s alphas of 0.90 and 0.79 for reciprocal filial piety and authoritarian filial piety, respectively (Yeh and Bedford, 2003). In this study, Cronbach’s alphas for reciprocal filial piety and authoritarian filial piety were 0.92 and 0.86, respectively.

### Procedure

Ethical clearance for the study was granted by the Human Research Ethics Committee of the Department of Social Work, Foshan University, China (Ref. 2020001). An online survey was administrated *via* wenjuanxin.cn. Participants were recruited using advertisements on Chinese social media (WeChat) and from the investigators’ professional, social, and personal contacts.

### Data Cleaning

Eleven participants were removed due to unengaged responding (i.e., selecting the highest/lowest possible value for all questions). Boxplots were used to identify extreme univariate outliers (i.e., those who scored three box lengths above or below the box boundary). These extreme outlying values were replaced with the next highest non-outlying values. Nine extreme univariate outliers were identified *via* this process (two on anxiety total, three on depression total, and four on reciprocal filial piety total). Mahalanobis distance figures were used to screen for multivariate outliers. Eight multivariate outliers were detected (using an α of 0.001; Tabachnick and Fiddel, 2013) and deleted, leaving 1,201 participants for the final analysis.

### Analysis

Data analysis was performed using IBM’s SPSS version 26. Analysis of variance (ANOVA) and Chi-square tests were employed to determine whether filial piety and demographic variables differed significantly across the three infection severity groups. General linear modeling (GLM) was used to investigate whether infection severity (RQ1), filial piety (RQ2), and their interaction terms (RQ3), would be predictive of mental health after adjusting for demographic differences between groups. Due to stress, anxiety, and depression scores being highly correlated (see below), a composite *mental distress* score was created by summing these scores (see section “Materials and Methods”). The RQs were then assessed *via* a single GLM (with this composite score being the outcome variable). Three predictor variables (infection severity, authoritarian filial piety, and reciprocal filial piety) were entered into this model, along with three control variables (age, gender, and highest level of educational attainment). Two product terms (infection severity × reciprocal filial piety and infection severity × authoritarian filial piety) were also entered to assess for an interaction between infection severity and filial piety. As advised by Hayes (2018), non-significant product terms were removed, and the model re-run, in order to increase interpretability of coefficient values.

## Results

### Preliminary Analysis

Analysis of variance indicated that the low [*M* = 40.66; 95% CI (40.24, 41.08); SD = 5.95], moderate [*M* = 41.23; 95% CI (40.29, 42.17); SD = 6.06], and high [*M* = 41.00; 95% CI (40.31, 41.69); SD = 5.88] infection severity groups did not differ in terms of reciprocal filial piety, *F*(2, 1,198) = 0.802, *p* = 0.448, η^2^ < 0.01. However, these groups did differ in regard to authoritarian filial piety, *F*(2, 1,198) = 5.11, *p* = 0.006, η^2^ = 0.01. Bonferroni corrected pairwise comparisons indicated that authoritarian filial piety was higher among the moderate infection severity group [*M* = 26.15; 95% CI (24.80, 27.50); SD = 8.69] compared with the low [*M* = 24.47; 95% CI (23.99, 24.95); SD = 6.77, *p* = 0.020] and high infection severity groups [*M* = 23.91; 95% CI (23.19, 24.78); SD = 7.07, *p* = 0.005]. The low and high infection severity groups did not significantly differ from each other in terms of authoritarian filial piety, *p* = 0.887.

Infection severity groups were found to differ in terms of age, *F*(2, 1,198) = 48.28, *p* < 0.001, η^2^ = 0.08, with the high infection severity group (*M* = 35.88; 95% CI [34.42, 37.34]; SD = 12.43) being significantly older than the low [*M* = 27.86; 95% CI (26.96, 28.76); SD = 12.64; *p* < 0.001] and moderate infection severity groups [*M* = 26.98; 95% CI (25.45, 28.50); SD = 9.84; *p* < 0.001]. The moderate and low infection severity groups did not significantly differ on age, *p* = 1. The infection severity groups were also found to differ in terms of gender, χ^2^ (2, *N* = 1,201) = 42.40, *p* < 0.001, Cramer’s *V* = 0.19, and highest level of education attainment, χ^2^ (8, *N* = 1,201) = 184.21, *p* < 0.001, Cramer’s *V* = 0.28. Accordingly, age, gender, and education were entered as control variables in the analysis of the research questions.

Zero-order correlations between continuous variables are reported in Table 3. As can be seen, the mental health indicators (stress, anxiety, and depression) exhibited large, positive correlations with one another. Reciprocal filial piety displayed moderate, negative correlations with all three mental health indicators, while authoritarian filial piety showed small negative correlations with stress and depression, but not anxiety. Reciprocal filial piety had a small, positive correlation with authoritarian filial piety.

**TABLE 3:** Zero-order correlations between continuous study variables.

	**Stress**	**Anxiety**	**Depression**	**RFP**	**AFP**	**Age**	**Gender^*a*^**
Stress		0.862***	0.847***	−0.216***	0.116***	−0.091**	0.098**
Anxiety			0.845***	−0.245***	–0.054	−0.108***	0.063*
Depression				−0.276***	−0.092**	−0.102***	0.036
RFP					0.107***	0.109***	0.055
AFP						0.088**	−0.241***
Age							0.029

^*a*^Point-biserial correlations with male = 0 and female = 1. All tests are two tailed; *p < 0.05; **p < 0.01; ***p < 0.001. RFP, reciprocal filial piety; AFP, authoritarian filial piety.

In order to examine the prevalence of stress, anxiety, and depression, participants were categorized according to DASS cut-points (Table 2). Table 4 reports on the percentage of participants falling into each category, for the overall sample and across the three infection severity groups. Table 4 also reports mean DASS scores for the overall sample and each severity group.

**TABLE 4:** Percentage of participants falling into each DASS category and means (standard deviations) for the overall sample and infection severity in region of residence.

**DASS category**	**Overall sample (*N* = 1,201)**	**Lowest infection severity (*n* = 758)**	**Moderate infection severity (*n* = 162)**	**Highest infection severity (*n* = 282)**
**Stress**				
Category				
Normal	79.1	80.6	85.8	71.2
Mild	8.2	7.3	4.9	12.4
Moderate	7.6	7.7	4.3	9.3
Severe	3.4	3.0	2.5	5.0
Extremely severe	1.7	1.5	2.5	2.1
Mean (SD)	8.40 (8.79)	8.09 (8.60)	6.54 (8.62)	10.32 (9.09)
**Anxiety**				
Category				
Normal	65.8	66.8	71.6	59.8
Mild	6.3	6.6	4.9	6.4
Moderate	14.5	14.1	10.5	17.8
Severe	6.1	5.7	6.2	7.1
Extremely severe	7.3	6.9	6.8	8.9
Mean (SD)	6.29 (7.44)	6.15 (7.28)	5.47 (7.99)	7.16 (7.50)
**Depression**				
Category				
Normal	71.0	71.8	77.8	65.1
Mild	11.3	11.9	5.6	13.2
Moderate	11.2	10.4	8.6	14.6
Severe	3.2	3.0	4.3	2.8
Extremely severe	3.3	2.9	3.7	4.3
Mean (SD)	6.38 (7.94)	6.20 (7.74)	5.30 (8.30)	7.52 (8.14)
				

Table 5 reports on the percentage of participants with elevated levels of stress, anxiety, and depression of any intensity (i.e., those categorized as displaying mild to extreme levels of stress, anxiety, or depression according to DASS cut-points). As can be seen, the highest infection severity group had a higher percentage of participants displaying elevated levels of stress (28.8%), anxiety (40.2%), and depression (34.9%), as compared with the moderate (stress: 14.2%; anxiety: 28.4%; depression: 22.2%) and low infection severity groups (stress: 19.4%; anxiety: 33.2%; depression: 28.2%).

**TABLE 5:** Percentage of participants displaying normal and elevated levels of stress, anxiety, and depression by infection severity of region of residence, along with adjusted standardized residuals for cells (in brackets).

**DASS category**	**Overall sample (*N* = 1,201)**	**Lowest infection severity (*n* = 758)**	**Moderate infection severity (*n* = 162)**	**Highest infection severity (*n* = 282)**
**Stress**				
Normal	79.1	80.6 (1.7)	85.8 (2.3)	71.2 (−3.7)
Elevated	20.9	19.4 (−1.7)	14.2 (−2.3)	28.8 (3.7)
**Anxiety**				
Normal	65.8	66.8 (0.9)	71.6 (1.7)	59.8 (−2.4)
Elevated	34.2	33.2 (−0.9)	28.4 (−1.7)	40.2 (2.4)
**Depression**				
Normal	71.0	71.8 (0.7)	77.8 (2.0)	71.0 (−2.5)
Elevated	29.0	28.2 (−0.7)	22.2 (−2.0)	29.0 (2.5)

Chi-square tests of contingencies were then used to formally assess whether infection severity was related to elevations in mental distress. A significant relationship was observed between infection severity and stress categorization, χ^2^ (2, *N* = 1,201) = 16.12, *p* < 0.001, Cramer’s *V* = 0.12. *Post hoc* testing was then conducted by generating adjusted standardized residuals, with residuals greater than two in absolute value being used to indicate a statistically significant deviation from the expected cell count under the null hypothesis (Agresti, 2013, p. 81). These adjusted standardized residuals are reported in brackets in Table 5. As can be seen, significantly more participants than expected were categorized as having elevated stress in the highest infection severity group. Furthermore, significantly fewer participants than expected were categorized as having elevated stress in the moderate infection severity group.

Anxiety categorization was also found to be related to infection severity, χ^2^ (2, *N* = 1,201) = 7.25, *p* = 0.027, Cramer’s *V* = 0.08. Again, *post hoc* testing revealed that significantly more participants than expected were categorized as having elevated levels of anxiety in the highest infection severity group. A similar pattern was found in regard to depression scores, χ^2^ (2, *N* = 1,201) = 8.55, *p* = 0.014, Cramer’s *V* = 0.08.

### Tests of RQs

As mentioned above, a GLM predicting a composite mental distress score was employed to assess RQs 1–3. In terms of RQ3, both product terms were non-significant predictors of mental distress—infection severity × reciprocal filial piety: *F*(2, 1,186) = 2.71, *p* = 0.067, $\eta_{p}^{2}=0.005$; infection severity × authoritarian filial piety: *F*(2, 1,186) = 0.80, *p* = 0.452, $\eta_{p}^{2}=0.001$—indicating a lack of interaction between infection severity and filial piety when predicting mental distress. Accordingly, these product terms were then dropped from the model. This final model was significant, *F*(10, 1,190) = 13.86, *p* < 0.001, accounting for about 10% of the variance in mental distress (*R*^2^ = 0.10). Parameter estimates are reported in Table 6.

**TABLE 6:** Parameter estimates for model predicting mental distress.

**Parameter**	***B***	**SE**	***P***	**$\eta_{p}^{2}$**
				
Intercept	72.20	5.24	<0.001	0.14
Lowest infection severity	−6.09	1.61	<0.001	0.01
Moderate infection severity	−6.26	2.31	0.007	0.01
Highest infection severity^*a*^				
Reciprocal filial piety	−0.92	0.11	<0.001	0.06
Authoritarian filial piety	−0.06	0.09	0.548	<0.01
Age	−0.16	0.06	0.004	0.01
Male	−2.76	1.38	0.046	<0.01
Female^a^				
Intermediate school	−5.69	4.22	0.177	<0.01
High school	−8.28	3.11	0.008	0.01
Diploma	−5.72	2.59	0.027	<0.01
Undergraduate	−0.87	1.71	0.608	<0.01
Postgraduate^a^				

*^*a*^Reference category.*

Regarding RQ1, the high infection severity group displayed significantly higher levels of mental distress than the low and moderate infection severity groups, even when controlling for the other variables in the model. Due to the coding scheme used, Table 6 does not provide information on the difference between the lowest and moderate infection severity groups, but further pairwise comparisons indicated a non-significant difference here, *p* = 0.932. Estimated marginal means (adjusting for all variables in the analysis) for the low, moderate, and high infection severity group were 16.89 [95% CI (14.28, 19.49); SE = 1.33], 16.71 [95% CI (13.09, 20.33); SE = 1.85], and 22.97 [95% CI (19.68, 26.26); SE = 1.68], respectively.

Regarding RQ2, reciprocal filial piety was a negative predictor of mental distress when controlling for the other variables in the model, with the *b*-value indicating that, holding constant all other variables, a one-unit increase in reciprocal filial piety was associated with a 0.92-unit decrease in mental distress. Conversely, authoritarian filial piety was not found to be predictive of mental distress. While only the findings in relation to the model predicting the composite outcome variable are presented here, the same analysis was carried out for all three mental health variables (stress, anxiety, and depression), with the same pattern of findings being observed in relation to each research question. These findings are provided in the Supplementary Material.

## Discussion

This study aimed to explore whether the degree to which one’s community is being impacted by COVID-19 (infection severity of region of residence) would be associated with mental health outcomes (stress, anxiety, and depression). The study also sought to assess whether higher levels of filial piety (both reciprocal and authoritarian) would be associated with better mental health during the COVID-19 pandemic, and whether infection severity and filial piety would interact in their association with mental health.

### Infection Severity and Mental Health

The data generally supported the notion that greater infection severity of region of residence would be associated with higher levels of mental distress. The highest infection severity groups displayed significantly more mental distress than the low infection severity group, even when statistically controlling for group differences in demographic variables (age, gender, and education) and filial piety. This is reflected in the preliminary analysis into the prevalence of stress, anxiety, and depression, where the high infection severity group had a significantly higher prevalence of elevated levels of stress, anxiety, and depression. The prevalence of elevated stress (28.8%), anxiety (40.2%), and depression (29.0%) found among the high infection severity group is similar to that reported by Wang et al. (2020; stress: 32.1%; anxiety: 36.4%; depression: 30.3%), which similarly utilized the DASS-21 among 1,210 participants from across China.

The findings suggest that those who were “closest” to the pandemic felt more mental distress. This is consistent with recent studies which have found that the rise in COVID-19 cases in China was associated with increased concern around contracting COVID-19 among the general public, as well as more general anxiety (Bao et al., 2020; Cao W. et al., 2020). The current findings are also consistent with the bulk of extant literature (e.g., Brooks et al., 2020; Dong and Bouey, 2020; Ho et al., 2020; Pfender, 2020; Rajkumar, 2020; Savage et al., 2020; Steingard, 2020; Wang et al., 2020; Xiao et al., 2020a,b; Zandifar and Badrfam, 2020; Zhou et al., 2020), much of which highlights the detrimental impact of the COVID-19 pandemic, and associated quarantine measures, on mental health.

### Filial Piety and Mental Health

Higher levels of reciprocal filial piety were associated with lower levels of stress, anxiety, and depression in the zero-order correlation analysis (Table 3). Additionally, greater reciprocal filial piety was associated with lowered levels of mental distress in the GLM (Table 6). In fact, reciprocal filial piety displayed the largest association with mental distress of any of the predictor variables assessed, according to the effect size measures computed. These findings are consistent with existing literature (Yeh, 2006; Leung et al., 2010; Chen, 2014; Chen et al., 2016; Jen et al., 2019) which indicates a positive relationship between reciprocal filial piety and psychological wellbeing.

Authoritarian filial piety was found to have a small, negative, zero-order correlation with stress and depression (Table 3). However, it was not found to be a significant predictor of mental distress in the GLM. The finding that higher levels of authoritarian filial piety were associated with less stress and depression is inconsistent with existing literature, much of which suggests that authoritarian filial piety has a negative impact on psychological wellbeing (Leung et al., 2010; Chen et al., 2016; Jen et al., 2019). This inconsistency may be due to differences in sample age. The studies listed above sampled Chinese adolescent and youth, while nearly half of the participants in the current study were over 25 years of age. Furthermore, the extant studies assessed wellbeing-related variables—happiness (Chen et al., 2016), life satisfaction, mental wellness (Jen et al., 2019), and psychological adjustment (Leung et al., 2010)—as opposed to stress, anxiety, and depression. Given that authoritarian filial piety was not a significant predictor of mental distress in the GLM, it is likely that the zero-order associations observed between authoritarian filial piety, stress, and depression are the result of shared variance with reciprocal filial piety, age, gender, and education. Hence, the data could be interpreted as suggesting that authoritarian filial piety is not associated with mental health in either direction during times of crisis, such as the COVID-19 pandemic outbreak.

### Lack of Interaction Between Infection Severity and Filial Piety in Predicting Mental Health

No interaction was found between infection severity and either filial piety variable in the analysis of the research questions. These findings indicate that the negative association between reciprocal filial piety and mental distress that was observed in the GLM was similar in magnitude for all infection severity groups; further suggesting a robust association between reciprocal filial piety and Chinese people’s mental health during crisis events. These findings also indicate that the observed lack of an association between authoritarian filial piety and mental distress was consistent across infection severity groups. That is, it was not the case that greater authoritarian filial piety was associated with better mental health among those in the low infection severity group, but not in the moderate or high infection severity groups (or vice versa).

### The Important Role of Reciprocal Filial Piety in Mental Health During the COVID-19 Pandemic

The negative relationship between reciprocal filial piety, a culture-specific worldview, and mental distress, suggests that reciprocal filial piety may positively influence Chinese people’s mental health during the COVID-19 pandemic. In other words, reciprocal filial piety may help Chinese people deal with the existential threats associated with pandemics. According to TMT, awareness of death increases the importance of cultural worldviews to reducing existential anxiety, fostering a range of activities to promote relevant cultural worldviews, such as reciprocal filial piety, which can decrease defensive reactions (e.g., mental distress) to death salience (Jonas et al., 2014). Traditionally, filial piety is manifested by co-residing with parents, providing material and emotional support to parents, and caring for parents (Li et al., 2010). During the pandemic quarantine, many Chinese people lived with, provided support to, and looked after, their parents. Reciprocally, many parents offered household support (e.g., cooking, cleaning, and looking after grandchildren) in return for their children’s filial behaviors. These activities affirm reciprocal filial piety. The affirmation of reciprocal filial piety provides Chinese people with the cognitive flexibility needed to adapt to the reality of the spread of COVID-19; helping them to conceive a future for their parents where COVID-19 is under control, sacrifice their personal freedoms and desires to comply with quarantine measures for the protection of their parents, and reflect upon their relationships with their parents during this time of crisis. This flexibility and adaptability may help Chinese people deal with uncertainty and feel significant when supporting their parents; allowing individuals to derive satisfaction within a cultural worldview framework, as suggested by TMT (Greenberg et al., 1986).

The relational nature of reciprocal filial piety suggests that reciprocal filial piety may assist individuals to maintain meaning in a way that lessens mental distress in the face of crisis. Applying reciprocal filial piety to the familial context, during the pandemic quarantine, people were likely to spend more time with their families. People may receive more support from their family members, actively re-evaluate the relational resources offered by their parents, and adopt flexible response strategies to deal with changing relations during this difficult time. The relational flexibility may enact relational mechanisms that provide harmonious and balanced benefits to individuals and their parents (Gopal and Koka, 2012). These relational mechanisms involve willingness, coordination, and collaboration in social interactions to adapt to changing circumstances (Malca and Bolanos, 2020). As proposed by MMM, through relational flexibility, people may respond to meaning violations by re-establishing coherent familial relations (Proulx and Inzlicht, 2012) and, thus, constructing a new meaning framework with their parents.

From a parallel filial piety perspective, a positive and reciprocal authority–citizen relationship may also play an important role in coping with the COVID-19 pandemic. Prior to the COVID-19 pandemic, the authority-citizen relationship might not have been as salient in Chinese people’s everyday lives as it was during the pandemic, when public health measures to control the spread of the virus became a norm. For example, the Chinese government provided updates on the latest pandemic developments, including infection and active case numbers on a daily basis *via* internet channels and social media (Bao et al., 2020). The frequent communication initiated by the government may promote new relational structures and a new meaning framework to compensate the interrupted relational system caused by the pandemic quarantine. When the authorities, a symbolic parental figure for Chinese people (Li et al., 2020c), interact with citizens rationally, affectionately, and attentively through consistent public communication, Chinese citizens may be more likely to perceive the government’s decisions as righteous, kind, fair, and benevolent. Chinese citizens may therefore develop positive emotional attachment to the authority-citizen relationship and behave reciprocally to demonstrate faithfulness, compliance, and obedience in response to the perceived caring, warm, and supportive nature of the authorities (Li et al., 2020c). People’s willingness to obey pandemic quarantine restrictions may result in the formation of new, coherent, and strong bonds with this symbolic parent. The establishment of new bonds leads to the creation of new relational structures in one’s meaning system, which may decrease tension, stress, and anxiety.

It is also worth noting that the lack of an interaction effect between reciprocal filial piety and infection severity could be interpreted as undermining the notion that filial piety uniquely guards against that kinds of existential threats experienced during pandemics (as the association between reciprocal filial piety and mental distress was not greater for those living in a more severely infected area). Rather, the results could be interpreted to indicate that reciprocal filial piety has a more generalized benefit to mental health among Chinese people.

### Limitations and Future Directions

There are a number of limitations of this study that should be noted. First, due to the cross-sectional nature of the study design, it cannot be determined with certainty whether higher levels of reciprocal filial piety caused better mental health. It is possible that being mentally healthy allows people to more thoroughly engage in, and draw more satisfaction, from filial relationships (be they literal or symbolic). One recent longitudinal study into the mental health of Chinese university students before, during, and after COVID-19 quarantine (Li et al., 2020c) found stress, anxiety, and depression to follow a V-shaped growth trajectory. That is, stress, anxiety, and depression decreased from before, to during, the quarantine period, before increasing again in the post-quarantine period. It is impossible to tell if a similar pattern of change took place among the current sample, given that the mental health of this sample was assessed only once during the COVID-19 pandemic. While the study of Li et al. (2020c) undermines the notion that quarantine measures are inherently distressing, the findings are not directly comparable with the current study. Those in the highest infection severity location displayed greater levels of mental distress in the current study; however, it is still possible that if the mental health of participants was measured at multiple points in time, a V-shaped change trajectory would be found.

Second, the instrument used to measure mental health in the current study (the Chinese language version of the DASS-21) was developed based on a Western measure and then translated, rather than being a measure that was specifically developed for use with Chinese samples. Furthermore, the cut-points which were used to categorize participants were generated based on the percentile distribution of scores in a non-clinical sample of Australians (Lovibond and Lovibond, 1995), as opposed to a Chinese sample. The popularity of the DASS as a research instrument allows for the findings of the current study to be directly compared with many other studies. However, it should also be recognized that Chinese models for comprehending mental distress symptoms or classifying depression, anxiety, and stress can be different from Western models. Accordingly, while DASS scores demonstrated high levels of reliability in the current study, the instrument may still not capture some culturally specific elements of mental distress.

Third, the sample is relatively highly educated and predominately employed in skilled occupations. Such a group may have more social resources to cope with the pandemic and be less likely to feel financial strain as a result of COVID-19 quarantine measures, compared with those in low-skilled occupations.

Fourth, although the reciprocal authority–citizen relationship offers a cultural framework for understanding social dynamics in Chinese culture, the current study did not explicitly test whether this relationship directly contributed to the maintenance of mental health among Chinese people during the COVID-19 pandemic. This is a possibility which warrants future research.

Finally, it should be recognized that the findings of this study may only be relevant in the Chinese context, as the authority–citizen relationship, which stems from the cultural concept of filial piety is unique to China. However, the findings still indicate the importance of reciprocal filial piety to maintaining mental health during pandemic crises. Future studies conducted with people from other cultures should consider assessing the mental health effects of worldviews indigenous to these cultures.

## Conclusion and Cultural Implications

In conclusion, the current study indicates that COVID-19 infection severity is positively associated with stress, anxiety, and depression. Furthermore, higher levels of reciprocal filial piety were found to be associated with better mental health among all infection severity groups, while authoritarian filial piety was found to have no relationship with mental health in any infection severity group (once accounting for demographic variables). Accordingly, the results of the present study highlight the importance of reciprocal filial piety to Chinese people’s mental health during times of crisis.

The findings of the current study provide relevant information regarding the design and implementation of mental health programs in response to COVID-19 and future pandemics. The present analysis suggests that, while being proximal to a pandemic can contribute to mental distress, cultural worldviews, such as reciprocal filial piety, may help minimize mental distress. When people feel they are being cared for and supported, and believe that their contributions will help to combat COVID-19, they are likely to build reciprocal relationships and maintain mental health. This has important ramifications for Chinese public health strategies during future infectious disease outbreaks, and other crises such as natural disasters and economic crises. The inclusion of strategies that buttress cultural resources, like reciprocal filial piety, within governmental responses the COVID-19 outbreak, may assist in mitigating some of the more negative consequences associated with these situations, potentially even increasing compliance with government initiatives.

## Data Availability Statement

The datasets presented in this study can be found in online repositories. The names of the repository/repositories and accession number(s) can be found below: The Research Data JCU (https://doi.org/10.25903/68CF-ZP16).

## Ethics Statement

Ethical clearance for the study was granted by the Human Research Ethics Committee of the Department of Social Work, Foshan University, China (Ref. 2020001). The patients/participants provided their written informed consent to participate in this study.

## Author Contributions

WL contributed substantially to the conception of the study, design of the analysis, and writing the major portion of the manuscript. YL and HY contributed substantially to the research design and data collection. DM contributed substantially to organizing and conducting statistical analysis, interpreting the results, and drafting the manuscript. CR contributed to drafting the manuscript and providing critical revision of the article. FY contributed to the acquisition of data. All authors contributed to the article and approved the submitted version.

## Conflict of Interest

The authors declare that the research was conducted in the absence of any commercial or financial relationships that could be construed as a potential conflict of interest.
