# Supplementary material for: Mental Health of Chinese People During the COVID-19 Pandemic: Associations With Infection Severity of Region of Residence and Filial Piety
Source: Front Psychol. 2021 May 28;12:633452. doi: 10.3389/fpsyg.2021.633452 (PMC8192824; doi:10.3389/fpsyg.2021.633452)
Supplement: Supplementary file 1 [file Data_Sheet_1.docx]

**Supplementary File to:**

**Mental health of Chinese people during the COVID-19 pandemic: Associations with infection severity of region of residence and filial piety**

**General Linear Model Predicting Stress**

For the first model predicting stress, both product terms were non-significant—infection severity × reciprocal filial piety: *F*(2, 1186) = 2.99, *p* = .051, η_p_^2^ = .01; infection severity × authoritarian filial piety: *F*(2, 1186) = 0.31, *p* = .737, η_p_^2^ < .01—indicating a lack of interaction between infection severity and filial piety when predicting stress. These product terms were then dropped from the model. After this, the overall model was significant, *F*(10, 1190) = 13.12, *p* < .001, *R*^2^ = .10. Parameter estimates are reported in Table S.1.

Table S.1

*Parameter Estimates for Model Predicting Stress*

| Parameter | B | SE | p | η_p_^2^ | |
| --- | --- | --- | --- | --- | --- |
|  |  |  |  |  |  |
| Intercept | 26.68 | 2.03 | < .001 | .13 | |
| Lowest Infection Severity | -2.64 | 0.62 | < .001 | .02 | |
| Moderate Infection Severity | -2.99 | 0.89 | .001 | .01 | |
| Highest Infection Severity^a^ |  |  |  |  | |
| Reciprocal Filial Piety | -0.29 | 0.04 | < .001 | .04 | |
| Authoritarian Filial Piety | -0.04 | 0.04 | .227 | < .01 | |
| Age | -0.06 | 0.02 | .005 | .01 | |
| Male | -1.38 | 0.53 | .010 | .01 | |
| Female^a^ |  |  |  |  | |
| Intermediate School | -2.15 | 1.62 | .185 | < .01 | |
| High School | -3.86 | 1.19 | .001 | .01 | |
| Diploma | -2.80 | 0.99 | .005 | .01 | |
| Undergraduate | -0.83 | 0.66 | .206 | < .01 | |
| Postgraduate^a^ |  |  |  |  | |
| a. Reference category | | | | |  |

As can be seen in Table S.1, the high infection severity group displayed significantly higher levels of stress than the low and moderate infection severity groups, even when controlling for the other variables in the model. Due to the coding scheme used, Table S.1 does not provide information on the difference between the lowest and moderate infection severity groups, but further pairwise comparisons indicated a non-significant difference here, *p* = .652. Estimated marginal means (adjusting for all variables in the analysis) for the low, moderate, and high infection severity group were 6.77 (95%CI [5.77, 7.77]; SE = 0.51), 6.42 (95%CI [5.03, 7.81]; SE = 0.71), and 9.41 (95%CI [8.15, 10.67]; SE = 0.64) respectively.

Table S.1 also shows that reciprocal filial piety was a negative predictor of stress when controlling for the other variables in the model. However, authoritarian filial piety was not found to be predictive of stress.

**General Linear Model Predicting Anxiety**

No evidence of interaction between infection severity and filial piety was observed in the model predicting anxiety—infection severity × reciprocal filial piety: *F*(2, 1186) = 1.78, *p* = .168, η_p_^2^ < .01; infection severity × authoritarian filial piety: *F*(2, 1186) = 1.82, *p* = .163, η_p_^2^ < .01. After excluding the interaction terms, the overall model was significant, *F*(10, 1190) = 11.30, p < .001, *R*^2^ = .09. Parameter estimates are reported in Table S.2.

Table S.2

*Parameter Estimates for Model Predicting Anxiety*

| Parameter | B | SE | p | η_p_^2^ | |
| --- | --- | --- | --- | --- | --- |
|  |  |  |  |  |  |
| Intercept | 21.24 | 1.71 | < .001 | .11 | |
| Lowest Infection Severity | -1.55 | 0.53 | .003 | .01 | |
| Moderate Infection Severity | -1.41 | 0.76 | .062 | < .01 | |
| Highest Infection Severity^a^ |  |  |  |  | |
| Reciprocal Filial Piety | -0.29 | 0.04 | < .001 | .05 | |
| Authoritarian Filial Piety | 0.02 | 0.03 | .561 | < .01 | |
| Age | -0.05 | 0.02 | .003 | .01 | |
| Male | -1.00 | 0.45 | .027 | < .01 | |
| Female^a^ |  |  |  |  | |
| Intermediate School | -1.75 | 1.40 | .205 | < .01 | |
| High School | -2.08 | 1.02 | .041 | < .01 | |
| Diploma | -1.52 | 0.85 | .073 | < .01 | |
| Undergraduate | -0.20 | 0.56 | .724 | < .01 | |
| Postgraduate^a^ |  |  |  |  | |
| a. Reference category | | | | |  |

The parameter estimates indicate significantly higher anxiety among the high infection severity group compared to the low infection severity group, but no difference between the moderate and high infection severity groups. Further pairwise comparisons indicated no difference between the low and moderate infection severity groups, *p* = .836. Estimated marginal means (adjusting for all variables in the analysis) for the low, moderate, and high infection severity group were 5.10 (95%CI [4.24, 5.95]; SE = 0.44), 5.23 (95%CI [4.05, 6.42]; SE = 0.60), and 6.65 (95%CI [5.57, 7.73]; SE = 0.55) respectively.

As above, reciprocal filial piety had a significant negative relationship with anxiety, while authoritarian filial piety was unrelated to anxiety.

**General Linear Model Predicting Depression**

Once again, both interaction terms were non-significant—infection severity × reciprocal filial piety: *F*(2, 1186) = 2.54, *p* = .079, η_p_^2^ < .01; infection severity × authoritarian filial piety: *F*(2, 1186) = 0.60, *p* = .548, η_p_^2^ < .01—indicating a lack of interaction. The overall model predicting depression was significant, *F*(10, 1190) = 13.81, *p* < .001, *R*^2^ = .10. Parameter estimates are reported in Table S.3.

Table S.3

*Parameter Estimates for Model Predicting Depression*

| Parameter | B | SE | p | η_p_^2^ | |
| --- | --- | --- | --- | --- | --- |
|  |  |  |  |  |  |
| Intercept | 24.28 | 1.81 | < .001 | .13 | |
| Lowest Infection Severity | -1.89 | 0.56 | .001 | .01 | |
| Moderate Infection Severity | -1.85 | 0.80 | .021 | < .01 | |
| Highest Infection Severity^a^ |  |  |  |  | |
| Reciprocal Filial Piety | -0.34 | 0.04 | < .001 | .06 | |
| Authoritarian Filial Piety | -0.03 | 0.03 | .344 | < .01 | |
| Age | -0.05 | 0.02 | .014 | < .01 | |
| Male | -0.40 | 0.48 | .419 | < .01 | |
| Female^a^ |  |  |  |  | |
| Intermediate School | -1.79 | 1.46 | .220 | < .01 | |
| High School | -2.35 | 1.08 | .029 | < .01 | |
| Diploma | -1.42 | 0.90 | .115 | < .01 | |
| Undergraduate | 0.15 | 0.59 | .797 | < .01 | |
| Postgraduate^a^ |  |  |  |  | |
| a. Reference category | | | | |  |

The parameter estimates indicate significantly greater depression among the high infection severity group compared to the low and moderate infection severity groups. Further pairwise comparisons indicated no difference in depression between the low and moderate severity groups (*p* = .955). Estimated marginal means (adjusting for all variables in the analysis) for the low, moderate, and high infection severity group were 5.02 (95%CI [4.12, 5.92]; SE = 0.46), 5.06 (95%CI [3.81, 6.31]; SE = 0.64), and 6.91 (95%CI [5.78, 8.05]; SE = 0.58) respectively.

Consistent with the above results, reciprocal filial piety was negatively associated with depression, whereas authoritarian filial piety showed no association with depression.
